# Heritable Targeted Inactivation of Myostatin Gene in Yellow Catfish (*Pelteobagrus fulvidraco*) Using Engineered Zinc Finger Nucleases

**DOI:** 10.1371/journal.pone.0028897

**Published:** 2011-12-14

**Authors:** Zhangji Dong, Jiachun Ge, Kui Li, Zhiqiang Xu, Dong Liang, Jingyun Li, Junbo Li, Wenshuang Jia, Yuehua Li, Xiaohua Dong, Shasha Cao, Xiaoxiao Wang, Jianlin Pan, Qingshun Zhao

**Affiliations:** 1 Model Animal Research Center, MOE Key Laboratory of Model Animal for Disease Study, Nanjing University, Nanjing, China; 2 Zhejiang Provincial Key Lab for Technology and Application of Model Organisms, School of Life Sciences, Wenzhou Medical College, Wenzhou, China; 3 Freshwater Fisheries Research Institute of Jiangsu Province, Nanjing, China; 4 College of Life Sciences, Nanjing Normal University, Nanjing, China; Auburn University, United States of America

## Abstract

Yellow catfish (*Pelteobagrus fulvidraco*) is one of the most important freshwater aquaculture species in China. However, its small size and lower meat yield limit its edible value. Myostatin (MSTN) is a negative regulator of mammalian muscle growth. But, the function of Mstn in fish remains elusive. To explore roles of *mstn* gene in fish growth and create a strain of yellow catfish with high amount of muscle mass, we performed targeted disruption of *mstn* in yellow catfish using engineered zinc-finger nucleases (ZFNs). Employing zebrafish embryos as a screening system to identify ZFN activity, we obtained one pair of ZFNs that can edit *mstn* in yellow catfish genome. Using the ZFNs, we successfully obtained two founders (Founder July29-7 and Founder July29-8) carrying mutated *mstn* gene in their germ cells. The mutated *mstn* allele inherited from Founder July29-7 was a null allele (*mstn^nju6^*) containing a 4 bp insertion, predicted to encode function null Mstn. The mutated *mstn* inherited from Founder July29-8 was a complex type of mutation (*mstn^nju7^*), predicted to encode a protein lacking two amino acids in the N-terminal secretory signal of Mstn. Totally, we obtained 6 *mstn^nju6/+^* and 14 *mstn^nju7/+^* yellow catfish. To our best knowledge, this is the first endogenous gene knockout in aquaculture fish. Our result will help in understanding the roles of *mstn* gene in fish.

## Introduction

Yellow catfish (*Pelteobagrus fulvidraco* Richardson) is a teleost fish belonging to Siluriformes, commonly found in Yangtze River, China. The increasing demand from national and international markets for this delicious freshwater fish promotes it becoming one of the most important freshwater farmed species in China. However, the small size and low amount of muscle mass limit its edible value. Myostatin (MSTN), a member of the transforming growth factor β superfamily, is a negative regulator of mammalian muscle growth [1]. *Mstn* knockout mice display 2- to 3-fold increase in both myofiber size (hypertrophy) and myofiber number (hyperplasia) than their heterozygous and wild-type littermates [2]. Mammals including cattle, sheep, dog, mouse and human beings with spontaneous mutations in their *Mstn* gene all exhibit double-muscle phenotype [1]. For example, the Belgian Blue cattle with an 11-bp deletion occurring in the third exon of *Mstn* that eliminates the entire bioactive domain of the protein exhibits 20–25% more muscle mass than standard breeds due to skeletal muscle hyperplasia [3]. However, no such mutations have been found in other vertebrates including fish [1]. To investigate roles of *mstn* gene in yellow catfish growth and create a strain of yellow catfish with increased muscle growth, it is necessary to knock out *mstn* [4] in yellow catfish.

Traditionally, creating a gene knockout animal is solely dependent on the availability of embryonic stem cell lines that have been only established in mouse and rat [5], [6]. Recently, zinc-finger nuclease (ZFN) technology has provided powerful tools for editing genomes of any animals [7]. Employing ZFN technology, researchers have accomplished gene knockout in domesticated animals such as silkworm (*Bombyx mori*) [8], pig (*Sus domestica*) [9], [10] and rabbit [11] in addition to model animals including fruit fly (*Drosophila melanogaster*) [12], zebrafish (*Danio rerio*) [13], [14], frog (*Xenopus tropicalis*) [15], mouse [16], [17] and rat [18]. However, no knockout farmed fish has been reported though zebrafish is the first vertebrate animal that its genome was edited with ZFN [13], [14]. In this study, we report targeted disruption of *mstn* gene in yellow catfish using ZFNs.

## Results and Discussion

**Figure 1:**
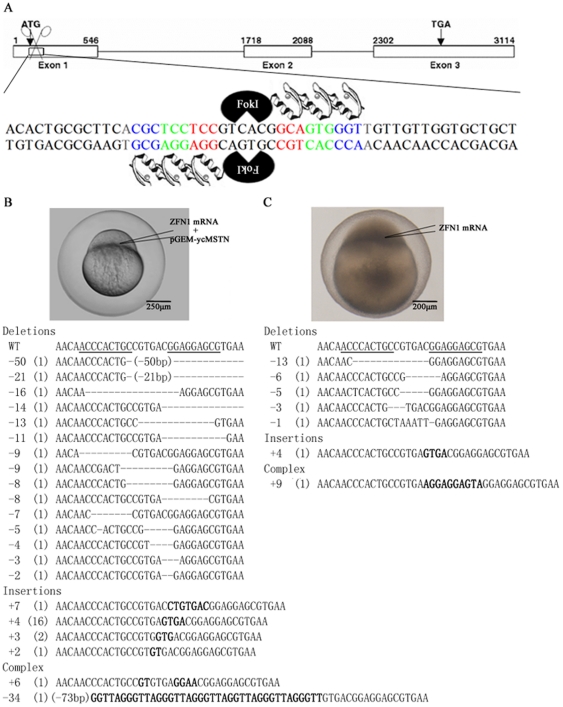
Zebrafish embryos can be used as an *in vivo* system to examine ZFN activity of editing yellow catfish *mstn* gene. (A) Schematic diagram shows ZFN1 binding to the yellow catfish *mstn* gene. Yellow catfish *mstn* exons are shown as boxes and its introns are shown as solid lines. Start codon (ATG) and stop codon (TGA) are marked in exon 1 and exon 3, respectively. Number above the exon box denotes the position of nucleotides in the gene [4]. The ZFN1 binding site is in exon 1. The triplets of nucleotides recognized by ZFN1 fingers are marked in different colors. (B) Zebrafish embryos were used as an *in vivo* system to examine ZFN activity of editing yellow catfish *mstn* gene. The plasmid containing exon 1 of yellow catfish *mstn* gene (pGEM-ycMSTN) was co-microinjected with ZFN1 mRNA into zebrafish embryos at 1–2-cell stage. The *mstn* molecules were amplified from the zebrafish embryos at 24 hpf and then subcloned for sequencing. Analyses on sequences of the molecules revealed that the molecules of disrupted *mstn* were categorized into three groups including deletions, insertions and complex. (C) ZFN1 cut *mstn* in yellow catfish genome. ZFN1 mRNA was microinjected into yellow catfish embryos at 1–2-cell stage. The *mstn* molecules were amplified from the yellow catfish embryos at 72 hpf and then subcloned for sequencing. Analyses on sequences of the molecules revealed that the molecules of disrupted *mstn* in yellow catfish genome were categorized into three groups including deletions, insertion and complex. WT: partial sequence of wild type *mstn* containing ZFN1 targeting site (B, C). Number in the leftmost of the panels (B, C) shows the number of nucleotides was deleted (−) or inserted (+) in the mutated *mstn* gene. Number in the bracket shows the frequency of the mutated molecules (B, C). Inserted nucleotides are bolded (B, C).

To knock out *mstn* gene (**DQ767967**) in yellow catfish, we designed ZFNs that can cut yellow catfish *mstn* using the modular assembly method [19], [20], [21]. Totally, two potential target sites and their corresponding ZFN pairs (ZFN1 and ZFN2) were selected (Figure 1A, Table 1). To test the activity of the two pairs of ZFNs, we first co-microinjected the plasmid carrying a genomic DNA fragment of yellow catfish *mstn* gene containing target site of a ZFN pair with mRNA of the ZFN pair into zebrafish embryos at 1–2-cell stage. Sequencing analyses on the molecules of the yellow catfish *mstn* gene that were PCR amplified from the microinjected embryos at 24 hpf (hours post fertilization) revealed that 37 of 144 molecules were mutated in ZFN1 co-microinjected embryos. The mutations of the *mstn* molecules were categorized into three groups including deletions (15 of 37; 40.5%), insertions (20 of 37; 54.1%) and complex type containing both deletions and insertions (2 of 37; 5.4%) (Figure 1B). However, none of 100 molecules were mutated in ZFN2 co-microinjected embryos.

**Table 1:** Zinc fingers used in ZFN1 (active) and ZFN2 (inactive)*.

Finger	Helix	Triplet	Reference Number	Modular Source
Left F1 of ZFN1	QSSHLTR	GGT	ZF12	SGMO
Left F2 of ZFN1	RSDALSR	GTG	ZF31	SGMO
Left F3 of ZFN1	QSGDLTR	GCA	ZF40	SGMO
Right F1 of ZFN1	RSDDLTR	GCG	ZF7	SGMO
Right F2 of ZFN1	QSGHLQR	GGA	ZF25	SGMO
Right F3 of ZFN1	QSGHLQR	GGA	ZF44	SGMO
Left F1 of ZFN2	TTGNLTV	AAT	ZF77	Barbas
Left F2 of ZFN2	QLAHLRA	AGA	ZF82	Barbas
Left F3 of ZFN2	RADNLTE	CAG	ZF91	Barbas
Right F1 of ZFN2	TSGELVR	GCT	ZF72	Barbas
Right F2 of ZFN2	RSDELVR	GTG	ZF66	Barbas
Right F3 of ZFN2	RSDELVR	GTG	ZF66	Barbas

*The table is modified from output of http://zifit.partners.org/.

To test whether ZFN1 could cut the *mstn* gene in yellow catfish genome, we microinjected mRNA of the ZFN pair into animal poles of yellow catfish embryos at 1–2-cell stage. Sequencing analyses on the molecules of the yellow catfish *mstn* gene that were PCR amplified from the microinjected yellow catfish embryos at 72 hpf revealed that 7 of 288 molecules were mutated. The mutations in yellow catfish *mstn* were categorized into three groups including deletions (5 of 7; 71.4%), insertion (1 of 7; 14.3%) and complex type containing both deletion and insertion (1 of 7; 14.3%) (Figure 1C). The results demonstrated that ZFN1 has the ability to cut genomic *mstn* in yellow catfish genome.

**Figure 2:**
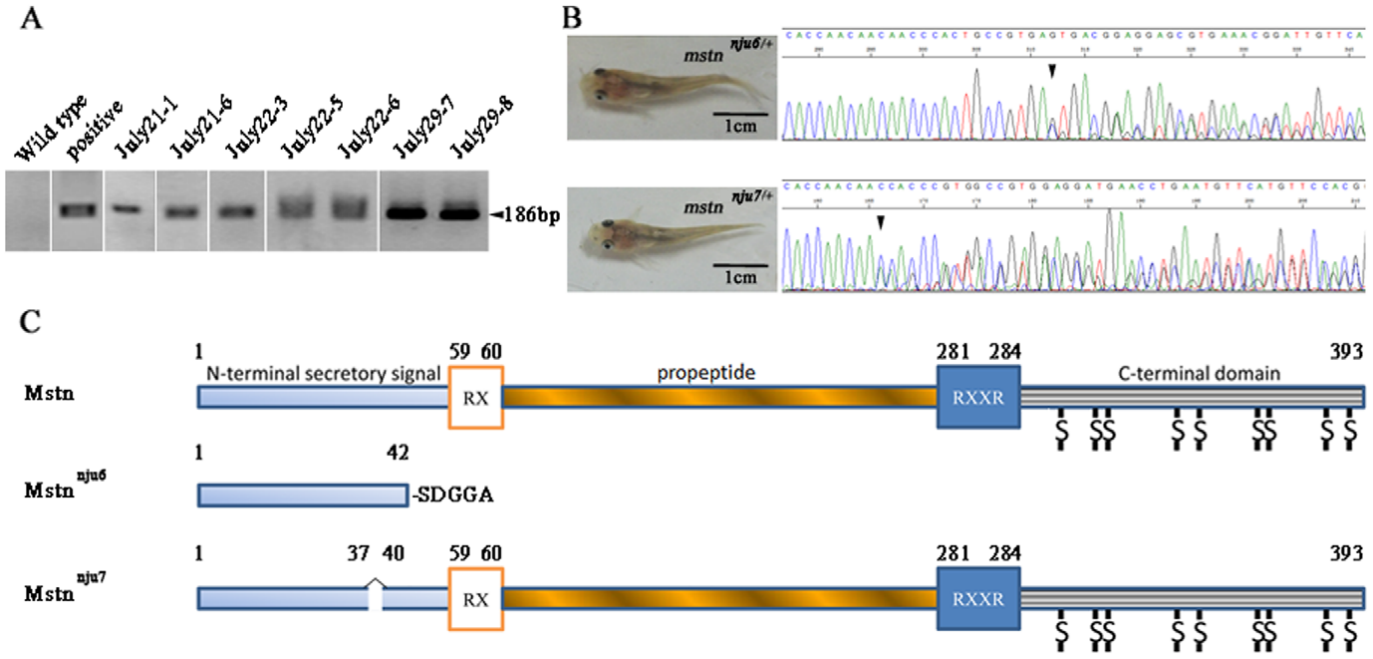
Generation of *mstn* knockout yellow catfish using engineered zinc finger nucleases. (A) 7 yellow catfish founders were identified to potentially carry mutated *mstn* in their germ cells by PCR. Agarose gel electrophoresis revealed that embryos from 7 yellow catfish founders gave out a predicted 186 bp product. The results suggested these fish carried a mutated *mstn* with the 4 bp insertion in their germ cells. “Wild type” and “Positive” denote the templates used for the 1^st^ round PCR were the genomic DNA isolated from a wild type yellow catfish (negative control) or the mutated *mstn* molecules with the 4 bp insertion (positive control), respectively. “July21-1, July21-6, July22-3, July22-5, July22-6, July29-7 and July29-8” denote the templates used for the 1^st^ PCR were the genomic DNA isolated from the different founders, respectively. (B) Sequencing chromatography shows the PCR products from two juvenile yellow catfish containing different mutated *mstn* alleles in their genomes. Arrow heads indicate that the mutated *mstn* alleles have different sequences from wild type allele stating from the base pointed. (C) Schematic diagram shows two mutated proteins would be produced from the two different strains of yellow catfish carrying different mutated *mstn* alleles. Mstn: Yellow catfish wild type Mstn comprises a signal sequence (N-terminal secretory signal), a propeptide domain (propeptide) and a bioactive domain (C-terminal domain). RX is a proteolytic site to remove the signal sequence and RXXR is a proteolytic processing site (RSSR) to produce bioactive form of Mstn. The number shows the position of amino acid residue [4]. Mstn^nju6^: A truncated protein encoded by *mstn^nju6^* contains only the 42 amino acid residues of the N-terminal secretory signal of yellow catfish Mstn plus 5 new amino acid residues. Mstn^nju7^: A mutated protein encoded by *mstn^nju7^* lacks two amino acid residues between aa37 and aa40 in the N-terminal secretory signal of yellow catfish Mstn.

To create *mstn* knockout yellow catfish, we microinjected more than 20,000 yellow catfish embryos at 1-cell stage with ZFN1 mRNA. When the microinjected embryos reached 2 months old, we selected founders that potentially carried mutated *mstn* alleles in their germ cells by genotyping each juvenile yellow catfish. The general strategy for us to select *mstn* knockout founders was to genotype all juveniles by sequencing the *mstn* molecules cloned from their fin genome as described above. Once it was identified to carry disrupted *mstn* gene in its somatic cells, a juvenile yellow catfish was selected as a founder. However, after analyzing the sequences of all mutated molecules created by ZFN1, we found that the molecule with the 4 bp insertion occurring most frequently (16 of 37; 43.2%) among the mutated molecules amplified from the zebrafish embryos that were used as a testing system was also present in the yellow catfish embryos microinjected with ZFN1 mRNA though at a lower frequency (1 of 7; 14.3%) (Figure 1B, 1C). Because the 4 bp insertion resulted in a null mutation of *mstn* gene, the yellow catfish carrying this type of mutation in their somatic cells had more chance to possess germ cells carrying the *mstn* null allele. Moreover, the screening of looking for the 4 bp insertion did not exclude other disruptions such as the other 6 six types of mutations we found (Figure 1C) because the frequency of the 4 bp insertion was only 14.3% of the mutated *mstn* molecules we detected from the yellow catfish embryos. In other words, a founder yellow catfish carrying the 4 bp insertion in its somatic cells must have many other types of disrupted *mstn* in its body. Furthermore, we found the 4 bp insertion could be specifically and easily picked out by two rounds of PCR (Figure 2A). Therefore, the strategy of looking for the 4 bp insertion was easier, less laborious, and more economical for us to identify a founder carrying disrupted *mstn* gene in their genome of somatic cells than the general strategy described above. Performing PCR that could identify the mutated *mstn* gene with the 4 bp insertion, we obtained 130 juveniles as founders from 577 juveniles examined.

When the founder yellow catfish reached 1 year old, 58 of the founders were used for artificial insemination by mixing reproductive cells of each founder yellow catfish with those of a wild type partner. PCR analyses on the embryos (F1) at 72 hpf produced from 31 founders revealed that 7 founders might produce embryos carrying mutated *mstn* allele (Figure 2A, Table 2). When the remaining offspring from the 7 founders reached 5 weeks old, we started to genotype each juvenile by directly sequencing PCR product amplified from its fin genome with primers specific for amplifying the *mstn* fragment containing ZFN1 targeting site. The results showed that some of the offspring produced from Founder July29-7 and Founder July29-8 carried mutated *mstn* gene (Figure 2B). Analyzing the DNA sequences of the molecules subcloned from the PCR product, we found the mutated *mstn* allele (*mstn^nju6^*) inherited from Founder July29-7 contains the 4 bp insertion, a microduplication of GTGA in the spacer (nt+244—+247) of ZFN1 targeting site (Figure 1C), predicted to encode a truncated protein only containing 42 amino acid residues of the N-terminal secretory signal of yellow catfish Mstn (Figure 2C) [4]. The result suggested that the mutated allele with the 4 bp insertion (*mstn^nju6^*) is a null allele. However, the mutated *mstn* (*mstn^nju7^*) inherited from Founder July29-8 was a complex type of mutation in which a 9 bp fragment (CAACCCACT, nt+228—+236) was replaced with a 3 bp fragment (ACA), predicted to encode a protein lacking two amino acid residues (aa38 and aa39) in the N-terminal secretory signal of yellow catfish Mstn (Figure 2C) [4]. The result was somehow surprising because the genotypes of the yellow catfish carrying disrupted *mstn* allele we screened from 156 offspring of Founder July29-8 were all *mstn^nju7/+^* but not *mstn^nju6/+^* though the PCR results from the genome of their sibling embryos at 72 hpf suggested that some of their siblings should carry *mstn^nju6^* allele (Table 2). But, the result that none of the 156 offspring carried *mstn^nju6^* allele is understandable if we consider that the germline transmission rate of *mstn^nju6^* allele could be as low as 0.5% (1/200) (Table 2). Additionally, germ cells and somatic cells of a founder do not necessarily share the same mutagenic genotype because the repair of a double strand break created by ZFN cutting in genomic DNA is an error-prone repair by non-homologous end joining and the repair occurring in the genomes of different cells would come out totally different results [7]. This was also evidenced in our observation that the sequences of most disrupted *mstn* molecules were unique not only in zebrafish embryos but also in yellow catfish embryos except that two molecules with the same +3 insertion were found in zebrafish embryos and the molecule with the 4 bp insertion were found not only in zebrafish embryos with much higher frequency (43.2%) but also in yellow catfish embryos with lower frequency (14.3%) (Figure 1B, 1C). Therefore, the offspring of Founder July29-8 carrying *mstn^nju7^* is explainable. Totally, we obtained 6 F1 yellow catfish carrying *mstn^nju6/+^* from Founder July29-7 and 14 F1 yellow catfish carrying *mstn^nju7/+^* from Founder July29-8 (Table 2).

**Table 2:** Summary of the identification of the yellow catfish carrying disrupted *mstn*.

Founder No.	Group(s) of embryos carrying mutated *mstn*/total examined	Number of juveniles carrying mutated *mstn*/total examined	Genotype of juveniles carrying mutated *mstn* (number of juveniles)
July21-1	1/8	0/51	N/A
July21-6	1/16	0/55	N/A
July22-3	1/16	0/30	N/A
July22-5	1/16	0/37	N/A
July22-6	1/16	0/196	N/A
July29-7	6/12	6/47	*mstn^nju6/+^*(6)
July29-8	1/20	14/156	*mstn^nju7/+^*(14)

In summary, we have created a strain of yellow catfish (*Pelteobagrus fulvidraco*) (*mstn^nju6/+^*) carrying an *mstn* null allele using ZFNs. To our best knowledge, this is the first endogenous gene knockout in farmed fish. It is known that ZFNs could cause off-target cleavage in organism genome. However, the off-target cutting could be separated from the desired mutation by backcrossing to the parent strain [18]. Therefore, we can obtain *mstn* null yellow catfish (*mstn^nju6/nju6^*) on a wild type background by genetic crossing. Provided we have yellow catfish homologous for *mstn* null allele (*mstn^nju6/nju6^*), we can investigate the roles of *mstn* gene in muscle growth of yellow catfish. Because Mstn is highly conserved during vertebrate evolution [1] and inhibiting the function of *mstn* either by knocking down *mstn* in zebrafish or overexpressing follistatin in trout results in increased growth in zebrafish or trout respectively [22], [23], it is very likely that piscine Mstn functions as a negative regulator of muscle growth like mammalian ones. However, unlike mammals, fish possess two copies of *mstn* gene or even four copies within salmonids likely due to tetraploidization [1]. To fully understand the roles of *mstn* gene in yellow catfish, we have to knock out the second *mstn* gene in yellow catfish. We are now looking for the other *mstn* gene in yellow catfish by deep sequencing the transcriptome of yellow catfish muscle. Once we find out a duplicated copy of *mstn* (*mstnb*) in yellow catfish, we will align its sequence with that of current *mstn* copy. If both genes share the same or highly similar sequence of ZFN1 target site, we will examine whether ZFN1 can also cut *mstnb* using the *in vivo* testing system we established. If ZFN1 can cut *mstnb*, we will examine the *mstn* knockout yellow catfish to see whether their *mstnb* is also disrupted. However, if *mstnb* is not disrupted in the *mstn* knockout yellow catfish, we will screen the other 62 founders we generated to look for the yellow catfish inheritably carrying *mstnb* null allele. If ZFN1 cannot cut *mstnb* gene, we will knock out the second *mstn* gene by designing new ZFNs or using the technology of the recently developed TALENs (transcription activator-like effectors nucleases) [24], [25].

## Materials and Methods

### Ethics statement

This study was approved by the Institutional Animal Care and Use Committee of Model Animal Research Center of Nanjing University under approved protocol (MARC-AP QZ01…Basic Zebrafish Protocol).

### Design of zinc finger nuclease targeting myostatin gene in yellow catfish

The ZFNs targeting yellow catfish *mstn* gene were designed using the modular assembly method [19], [20], [21]. Briefly, nucleotide sequence of the 1^st^ exon containing initiation codon of yellow catfish *mstn* gene [4] served as an input to search the targeting sites and their corresponding 3-zinc-finger left array and 3-zinc-finger right array using ZiFiT software (ZiFiT:http://zifit.partners.org/) [19], [20], [21]. Selected potential target sites in the yellow catfish *mstn* gene output from the software were aACCCACTGCCGTGACGGAGGAGCGt (nt+113—+138) (with GNN score 0.59 by 0.59 and affinity score 3.77 by 4.77) and gATTTCTCTGGGCTTCGTGGTGGCTt (+21—+46) (with GNN score 0.59 by 0.00 and affinity score 6.79 by N/A). The fingers in the output from the same modular sources (SGMO or Barbas) were selected to make the 3-zinc-finger arrays of two pairs of ZFNs correspondingly (Table 1). The sequences of 3-zinc-finger arrays were then inserted into the sequences of ZFN backbone [26], [27] to design two pairs of ZFNs (ZFN1 and ZFN2). The designed ZFNs were synthesized and then cloned into plasmids containing nuclear localization signal sequence under T7 promoter direction by a commercial company.

### Examination of ZFN activity in zebrafish embryos

The ZFN activity was examined first in zebrafish embryos. To perform the test, we cloned genomic DNA fragment of yellow catfish *mstn* gene containing 1^st^ exon into pGEM-T easy vector (Promega, USA) with primers CAAGGTGTTCCTGTTCCTGCTG (forward) and TCCTTGCTTGCTGCTATTCTGG (reverse) [4] using the genomic DNA isolated from yellow catfish fin as template. The resulting plasmid (pGEM-ycMSTN) was treated with proteinase K to make it RNase free. We next *in vitro* synthesized the corresponding mRNA (capped and tailed) of the ZFNs using as templates the ZFN plasmids with mMessage mMachine T7 Ultra Kit (Ambion, USA). The mRNA was then purified using Oligotex mRNA Mini Kit (Qiagen, Germany). The left arm and right arm mRNA of each ZFN pair was then mixed with plasmid pGEM-ycMSTN. The final concentration in the mixture was 200 ng/µl mRNA of each arm and 50 ng/µl of pGEM-ycMSTN. 1 nl of the mixture was microinjected into zebrafish embryos at 1–2-cell stage. The injected embryos were grown at 28.5°C and collected at 24 hpf to extract DNA. The extracted DNA was dissolved in 100 µl water and 1 µl of the DNA solution was then used as template to amplify the *mstn* fragment containing the potential ZFN targeting site by PCR using primers of GATCCCAAGGTGTTCCTGTT (forward) and CTTGAAGACGGAGCTGCTTG (reverse) in a 20 µl reaction system. The PCR conditions were 94°C for 2 min, 30 cycles of (30 s at 94°C, 30 s at 60°C, and 1 min at 72°C), and a final extension of 6 min at 72°C. The PCR product was then cloned into pGEM-T easy vector (Promega, USA) and transformed into DH5α competent cells. The transformants were randomly selected and identified by PCR as described above. 144 and 100 of the PCR positive transformants were further sequenced for examining activity of ZFN1 and ZFN2, respectively. The sequences were then aligned with the wild type sequence of yellow catfish *mstn* to determine whether they were mutated.

### Artificial insemination of yellow catfish

To obtain fertilized eggs of yellow catfish, we performed artificial insemination on yellow catfish. Two years old yellow catfish living in wild environment or one year old ones living in lab aquarium like zebrafish were used for artificial insemination. To carry out artificial insemination, we executed a first round of injection with 0.3 ml of 0.68% NaCl solution (sterilized) containing 3 µg of LHRH-A2 (Ningbo Sansheng Pharmaceutical Co., Ltd., Ningbo, China) into each female yellow catfish at 9:00 am, then a second round of injection with 0.3 ml of the solution containing 6 µg of LHRH-A2 and 300 IU hCG (Ningbo Sansheng Pharmaceutical Co., Ltd., Ningbo, China) into the same female yellow catfish at 11:00 pm and only one round of injection with 0.2 ml of the solution containing 4 µg of LHRH-A2 and 200 IU hCG into male yellow catfish at 11:00 pm the day before artificial insemination was performed. The injection was performed abdominally under pectoral fin. In the next morning, each injected female fish was examined for the maturity of its eggs. If eggs came out from apertura cloacalis of a female yellow catfish immediately after its abdomen was gently pressed, the eggs were recognized to be good for *in vitro* fertilization. We then collected the mature eggs by pressing the female yellow catfish hard in the abdomen and placed them into 100 mm culture dishes. Immediately after egg collection, the two entire testes of a male yellow catfish were taken out surgically and minced with scissors thoroughly. 3 ml of 0.69% NaCl was then added to suspend the minced testes. The solution containing sperms was immediately transferred to the dishes containing eggs and mixed thoroughly with the eggs. Shortly after the mixture, the fertilized eggs were dispersed into 100 mm culture dishes filled with aerated water. The eggs were changed with aerated water 1 minute later and were then ready for further experiment.

### Examination of ZFN activity in yellow catfish embryos

To test whether the ZFN pair that was able to cut yellow catfish *mstn* gene in zebrafish embryo system could cut *mstn* gene in yellow catfish genome, we microinjected 1 nl of the mixture containing 200 ng/µl mRNA of ZFN1 each arm into animal poles of yellow catfish embryos at 1–2-cell stage. The injected embryos were then grown at 28.5°C in the same conditions as zebrafish embryos. When reaching 72 hpf, 40 of the injected embryos were randomly selected to examine mutated *mstn* in their genomes by using the same procedures as described in the section of “*Examination of ZFN activity in zebrafish embryos*”. Totally, 288 of the PCR positive transformants were further sequenced for examining ZFN1 activity in yellow catfish embryos.

### Generation of heritable targeted inactivation of myostatin gene in yellow catfish

To generate *mstn* knockout yellow catfish, we first microinjected 1 nl of the mixture containing 200 ng/µl mRNA of ZFN1 each arm into animal poles of yellow catfish embryos at 1-cell stage. We then raised the injected embryos at 28.5°C in the same conditions as growing zebrafish embryos. When the yellow catfish reached 2 months old, a piece of tail fin was clipped from each juvenile and then submerged into 8.8 µl of the Trace DNA Extraction Solution to extract its genomic DNA using Trace DNA Extraction Kit (Nanjing Runbang Bio-tech Company, Nanjing, China). 1 µl of the extracted DNA was then used as PCR template directly to amplify the *mstn* fragment containing ZFN1 targeting site using the methods as described in the above section of “*Examination of ZFN activity in zebrafish embryos*” with a modification of reducing the cycle number to 20. A second round PCR was further performed using 1 µl of the 1^st^ round PCR product as template. The sequences of primer pair for the 2^nd^ round PCR were CCAACAGTCCAACAGCTCCTCT (forward) and CGCTTCACGCTCCTCCGTCACTCAC (reverse). The PCR conditions were 94°C for 2 min, 30 cycles of (30 s at 94°C, 30 s at 70°C, and 30 s at 72°C), and a final extension of 5 min at 72°C. The PCR products were then subjected to 1.5% agarose gel electrophoresis separation. The yellow catfish which produced a 186 bp PCR product were selected as founders to grow at 28.5°C in lab aquarium like zebrafish.

After having been raised for 10 months, the founder yellow catfish were transferred into a fishnet set in a wild pond starting from middle May of 2011 when the temperature was above 22°C in Nanjing, China. Two months after they had been raised in the pond, the founder yellow catfish were used for artificiality insemination by mixing reproductive cells of a founder yellow catfish with those of a wild type partner collected from local market. To test whether offspring (F1) of the founders contained mutated *mstn* gene, 80 to 200 embryos at 72 hpf produced from each founder were randomly selected for genotyping. Briefly, each 10 embryos were grouped to submerge into 88 µl of the Trace DNA Extraction Solution for extracting their genomic DNA using Trace DNA Extraction Kit (Nanjing Runbang Bio-tech Company, Nanjing, China). The extracted DNA was then used as PCR template to perform two rounds of PCR as described above. Once the embryos gave a PCR product sized 186 bp in the gel electrophoresis, the remaining F1 yellow catfish from the founder were raised in lab aquarium in the way similar to growing zebrafish for further screening.

When the offspring reached 5 weeks old, the tail fin of each F1 was clipped to amplify the *mstn* fragment containing ZFN1 targeting site as described above. The PCR product from each F1 was then subjected to sequence directly. The PCR product with mutated *mstn* molecules revealed by the sequencing chromatography was further subcloned to identify exact genotype for the individual F1. The F1 yellow catfish with mutated *mstn* gene were grown in lab aquarium in the way similar to growing zebrafish.
